# Self-Embedding Authentication Watermarking with Effective Tampered Location Detection and High-Quality Image Recovery

**DOI:** 10.3390/s19102267

**Published:** 2019-05-16

**Authors:** Chin-Feng Lee, Jau-Ji Shen, Zhao-Ru Chen, Somya Agrawal

**Affiliations:** 1 Department of Information Management, Chaoyang University of Technology, Taichung 41349, Taiwan; lcf@cyut.edu.tw; 2 Department of Management Information Systems, National Chung Hsing University, Taichung 40227, Taiwan; jjshen@nchu.edu.tw (J.-J.S.); g105029023@smail.nchu.edu.tw (Z.-R.C.)

**Keywords:** wireless sensor networks, image stenography, image authentication scheme, tamper detection, fragile watermarking method, self-recovery

## Abstract

Recently, sensor networks have emerged as a high-impact research area, and a number of high profile applications have been proposed. Although significant progress has already been made on securing basic network protocols, additional research is needed to produce techniques and methods for protecting canonical tasks in wireless sensor networks. In this paper, we propose an effective self-embedding authentication watermarking method for tampered location detection and image recovery. The proposed detection method is classified into block-wise and pixel-wise. In block-wise detection, if the size of the block is small, the false positive rate (FPR) will be low. In pixel-wise detection, when the tampered pixels are detected, only the corresponding pixel area is marked. Therefore, the FPR will be lower than that of the block-wise detection. The experimental results demonstrate that the proposed method was effective, and accurate tamper detection and high-quality recovery can be realized even in highly tampered images.

## 1. Introduction

Recently, sensor networks have emerged as a high-impact research area and a number of high profile applications have been proposed. Sensors are usually distributed in a sensory field and are used for applications, such as smart home, environmental monitoring, battlefield surveillance, information collection, etc. [1]. Wireless sensor networks (WSN) are distributed embedded systems where each unit is equipped with a defined amount of computation, communication, storage, and sensing resources. Such sensor networks have the capacity to store information not only about one or more users but they also contain a great deal of information about their past and even future actions. Moreover, once the sensors have been equipped with actuators, both the sensors and the environment can be impacted in a number of ways. However, WSNs are highly prone to security attacks intrinsically due to their deployment, their hardware, and their resource constraints. They are often deployed in uncontrolled and sometimes even hostile settings. The wireless communication networks on a large scale can be easily observed and interfered with. It is possible to manipulate the sensor networks even without interfering with the electronic subsystem of the node and actuators which can pose strong safety and hazard concerns. In addition, they have constraints in terms of energy and, therefore, extensive on-line security checking is not feasible. Therefore, wireless sensor network (WSN) nodes are complex component systems with numerous weak points from a security point of view. The role of security in WSN is highly important, and a number of security and privacy issues need to be addressed, such as how to ensure the integrity of sensor data, how to provide mechanisms for authentication and access control [2]. There is an urgent need to develop methods that ensure privacy of subjects and objects in the sensor networks. With watermarking techniques, the image or even video can be protected. We can detect the precise location of the tampered image or which frame of the video was damaged. Other methods, such as encryption, are unable to do so. The encryption method can only know that the file has been tampered rather than detect the damaged location.

Although significant progress has already been made in securing basic network protocols, additional research is needed to produce techniques and methods for protecting canonical tasks in wireless senor networks, such as routing, broadcast, multicast, and data aggregation. WSN require new concepts, techniques, and methods with respect to security, privacy, digital rights management, and usage measurement [3]. The Internet has been a great facilitator of computer and communication security on a large scale. However, it has itself created opportunities for new types of attacks, such as denial of service (DoS) and intrusion detection. In sensor networks, watermarking and other intellectual property protection techniques can be used at a variety of levels. Software used in the network and the design of sensor nodes can be protected using functional techniques. Both static and functional watermarking techniques [4] can be applied to the data collected from the network depending on the types of sensors and actuators deployed (i.e., video, audio, measured data).

In particular, image authentication technology is classified into two main types: the digital signature-based method and the watermarking method. Digital signatures are always stored by third parties in digital signature-based methods. In this approach, the digital signature retrieved from an image is compared with the digital signature stored by the third party. Comparing the two signatures helps us to detect whether the image has been tampered with or not [5,6,7,8]. The watermarking method can be categorized into robust watermarking [9,10,11,12,13,14,15], semi-fragile watermarking [16,17,18,19] and fragile watermarking [20,21,22,23,24,25,26,27,28,29,30,31,32,33,34]. In the robust watermarking method, hidden watermarks can be retrieved from the watermarked images after they have undergone image processing, such as noise processing or image compression. It can be used to validate copyrights and intellectual property rights. Fragile watermarks are hidden watermarks in the image that can be easily destroyed by tampering, and, thus, can also be accurately detected. Currently, there are two types of fragile watermarking technologies. The first type can only detect tampered digital images and locate the tampered areas. On the other hand, the second type can not only detect and locate the tampered areas, but it can also recover damaged areas in the image. The second type of image authentication technology can detect and locate tampered areas as well as do a self-recovery of the tampered areas, which makes it extremely helpful for the protection of image integrity. The present study utilizes this image authentication technology and proposes an effective self-embedding watermarking method that can not only detect an image’s tampered area accurately, but it can also improve image recovery capabilities of the tampered area effectively.

The remainder of this paper is organized as follows: In Section 2, we will introduce several methods that have been proposed in previous studies. Section 3 describes the proposed method that is classified as block-wise detection and pixel-wise detection. Experimental results and comparisons will be presented in Section 4 to demonstrate that the proposed method is more effective and better than other methods. Finally, Section 5 includes the conclusion of this paper.

## 2. Related Work

To understand the development and application of today’s fragile watermarking technologies, this section provides a brief on fragile watermarking technologies proposed by various scholars in the spatial, compression, and frequency domains in the recent years. Two types of data are generated by a sensor network: raw sensor data and processed application data. The aim here is to watermark all data provided by wireless sensor networks. The first type of sensor data is the original data the sensor network measures and the second type, processed data, is the output of the network. The second type of data is the information the user of the network expects from the network. The distinction of these two types of data [35] gives us a hint on where watermarking can take place: (i) during the process of sensing data (original data capturing); (ii) during the process of processing the original data. Since most image tampering detection methods are based on image blocks and ignore characteristics of the image blocks, it results in poor image quality after hiding the watermark. Therefore, in 2011 Lee et al. [16] proposed a semi-blind watermark scheme exploiting a self-reference image by using just noticeable distortion (JND) approach for digital image protection and authenticity. Hsu and Tu [23] in 2016 proposed adaptive embedding rules for the detection and recovery of image tampering that apply different hiding, detection, and recovery methods according to the block’s level of smoothness. The method mentioned above is implemented in the image’s spatial domain during the watermark generation process. If the recovery information is generated in the frequency domain, the quality of the recovered image will be much higher. In 2014, Lo and Hu [27] proposed a new reversible image authentication method which uses pseudo-random numbers to generate authentication data to serve as watermarks and uses a prediction-based histogram shifting scheme to embed the watermark into the original image. The complete watermark can be retrieved if the watermarked image has not been tampered with, and the watermarked image can be restored to the original image. In 2016, Singh and Singh [29] proposed an effective self-embedding watermarking method for image tamper and recovery capability positioning. This method uses Discrete Cosine Transformation (DCT) to generate authentication and recovery data from the image. During the embedding process, authentication data will be embedded in the block itself, and recovery bits will be embedded in mapping blocks, which increases the performance of image tamper detection. Because DCT is used to generate recovery data, the image quality will be better than the previous methods used in the past. Moreover, some studies have found that original images were unrecoverable if irreversible data hiding methods were used to embed watermarks. Therefore, reversible data hiding methods are used mostly to embed watermarks. The function of this method is that the original image can be recovered if the watermarked image has not been tampered with. However, it can only locate the tampered area and cannot recover the image. 

In 2016, Yin et al. [30] proposed a method to improve Lo and Hu’s approach [27]. It uses a Hilbert Curve to scan the entire image and allows neighbor pixel values to group together. Pixel value ordering (PVO) is then used to embed the watermark. This method enables better control of the changes in pixel value and watermark can be embedded according to the block’s complexity as well.

In 2017, Qin et al. [31] proposed a fragile image watermarking method with pixel-wise recovery based on overlapping embedding strategy. First, the original image $I_{O}$ (with a size of w×h) is divided into overlapping blocks B with a size of 3×3. Every block $B_{m,n}\;\left(m=1,2,\ldots ,h/2,\;n=1,2,\ldots ,w/2\right)$ should consist of three pixels that overlap with neighboring blocks. Then the embedding rule is applied which requires that every center pixel of each block should contain the authentication data A, and either one or two bits of the recovery data needs to be embedded in the pixel. The complexity level of the block is calculated, and two thresholds levels are used to determine whether the block is complex, general or smooth. Two to four bits of authentication data have different authentication data A according to their degree of complexity. The authentication data A is then generated by using the Hash function of the pixel value, block number, as well as the image’s name or ID. Its length is set according to the degree of complexity of the block. The generation of the recovery data R is then calculated by averaging the pixel value of M and its six most significant bits as $M^{\prime }$, and further combined with the entire $M^{\prime }$ image as U with a length of 6×(h×w/4). Thereafter, U is then scrambled and divided into k sub-sets by using the SK secret key. Each set contains four bits of recovery data R for each block. Authenticated and recovery data are then embedded in each block.

In 2018, Tai and Liao [33] proposed an efficient block-based fragile watermarking scheme for image tamper detection and self-recovery. In their method, the authentication and the recovery information were both generated by using the wavelet transform rather than the common block average method. The 4-bit authentication code was generated by using the low-frequency sub band of Haar wavelet transform of each block, and the 28-bit recovery code was produced by the low-frequency sub band and two high-frequency sub bands. To break the independency of each block the method used block mapping technique called Arnold’s cat map transform to scramble the image blocks. The authentication code and recovery code were then embedded from other blocks into each block.

Each of the five fragile watermarking techniques discussed above has their own advantages and disadvantages. The two methods of Lo and Hu [27] and Yin et al. [30] made use of reversible data hiding techniques to embed a watermark into the image so that the watermarked image can be restored to original image when no tampering occurs in the image. However, these two methods do not provide any recovery scheme to restore the tampered images, which means that they do not have the ability to recover the original image after the image has been tampered. The method proposed in this paper has designed a recovery scheme using mean value and block enlarger techniques to resolve this issue. In another method proposed by Singh and Singh [29], they used DCT (Discrete Cosine Transformation) to generate authentication and recovery information. The advantage of using DCT is that the recovered image quality will better as compared to other methods which just use the mean value to restore the original image. The methods proposed by Hsu and Tu [23] also have the advantage of better image quality of the recovered image as their methods use different procedures to recover information based on the level of smoothness of the block in the image. However, the experimental results of our proposed method have shown better performance in terms of image quality of the recovered image when compared to Singh and Singh’s [29] and Hsu and Tu’s [23] methods. The method of Qin et al. [31] can only detect the tamper when the tamper ratio is below 45%, and the proposed method not only enhances the recover image quality than the previous studies but also resolve the tamper detection issue of the Qin et al. [31] method. The method proposed by Tai and Liao have better recovery performance compared to other methods. However, when dealing with highly complex images their peak signal to noise ratio (PSNR) of recovered image is not quite good. Our proposed method overcomes this limitation.

## 3. Proposed Method

The image authentication technology proposed by our method can detect and locate tampered area as well as recover the tampered area. To detect tampered areas more accurately and improve their recovery effectively, our method proposes image authentication technology based on block-wise and pixel-wise detection methods. According to the experimental results, the error rate of the pixel-wise method is lower than the block-wise method. The quality of images recovered from using either pixel-wise or block-wise image authentication method is higher compared to other methods using fragile authentication method.

### 3.1. Description of Symbol Definitions

This section defines and describes all the symbols used in this paper.

1. W: the weight of the original image
2. H: the height of the original image
3. $I_{O}$: the original image
4. $I_{W}$: the watermarked image
5. $I_{T}$: the tampered image
6. $I_{R}$: the recovered image
7. m×n: the size of a block
8. N=(H×W)/(m×n): the total number of the blocks in the image
9. $B_{i}\left(i=1,2,\ldots ,N\right)$: each block of the image
10. $P_{j}^{i}\left(j=1,2,\ldots ,n\times m\right)$: the pixel value of each block
11. SK: the secret key
12. $rm_{i}$: the pseudo- random number that is generated by SK
13. $BM_{i}$: the mapping block
14. $M_{i}$: the mean value of each block
15. $R_{i}$: the recovery data of each block
16. $A_{i}$: the authentication data of each block
17. ${R^{\prime }}_{i}$: the recovery data that is generated from $I_{T}$
18. ${A^{\prime }}_{i}$: the authentication data that is generated from $I_{T}$
19. k: the size of the authentication data
20. q: the number of bits that will be embedded into each pixel
21. $T_{i}$: the table that is marked whether it has been tampered with
22. $TM_{i}$: the mapping table of $T_{i}$
23. L: the enlarged image

### 3.2. Block-Wise Detection

**Figure 1:**
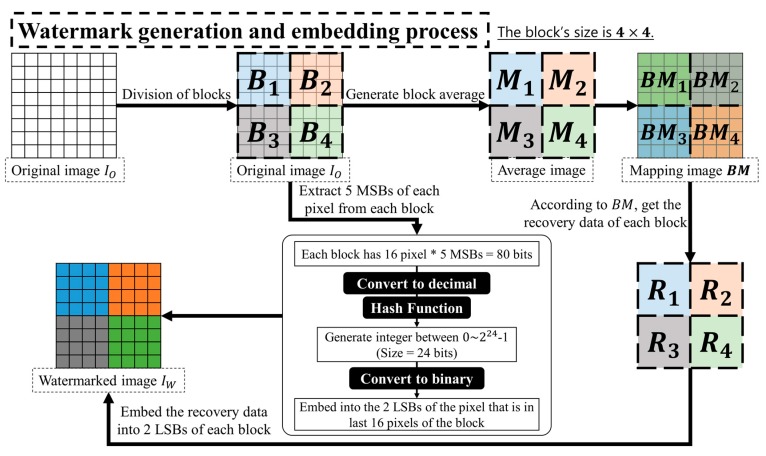
Flowchart of watermark generation and embedding process based on “block-wise detection” (The size of a block is 4×4).

First, the original image $I_{O}$ with a size of H×W is divided into N number of non-overlapping blocks $B_{i}\left(i=1,\;2,\ldots ,N\right)$ of m×n sizes. Recovery data, $M_{i}$, is the mean value of each block $B_{i}$, and the authentication data is created by encrypting the block information. The embedding process uses the least significant bit (LSB) replacement method to embed every *q* number of LSB pixel values in each block, which combines the *k*-bits authentication data $A_{i}$ and recovery data for mapping block $BM_{i}$. The flowchart of watermark embedding process is shown in Figure 1. The following steps provide a detailed description on watermark generation and embedding process.

Step 1. The original image $I_{O}$ is divided into N non-overlapping blocks $B_{i}\left(i=1,\;2,\ldots ,N\right)$. The size of each block $B_{i}$ is m×n.

Step 2. Using Equation (1) calculate the 8-bit mean value $M_{i}$ of each block as this block’s recovery data.

(1) $$M_{i}=\mathrm{round}\left(\left(\overset{m\times n}{\underset{j=1}{\sum }}P_{j}^{i}\right)/\left(m\times n\right)\right)$$

Step 3. The N pseudo-random numbers $rm_{i}\left\{1,\;2,\;\ldots ,\;N\right\},\;i=1,\;2,\ldots ,N$ are generated using the secret key SK. According to $rm_{i}$, the mapping block $BM_{i}$, that corresponds to block $B_{i}$, is created. The relationship between the original block $B_{i}$ and the mapping block $BM_{i}$ is shown in Equation (2). And the recovery data $R_{i}$ of the block $B_{i}$ is the mean value $M_{rm_{i}}$ of the mapping block $BM_{i}$, as shown in Equation (3). Table 1 shows the relationship between the original block and the mapping block.

(2) $$BM_{i}=B_{rm_{i}}$$

(3) $$R_{i}=M_{rm_{i}}$$

Step 4. The k-bit authentication data $A_{i}$ of each block $B_{i}$ is generated by Equations (4) and (5).

(4) $$a_{5\times \left(j-1\right)+x}=\mathrm{mod}\left(P_{j}^{i}/2^{8-\left(x-1\right)},2\right),\;x=\left(1,\;2,\ldots ,\;5\right)$$

(5) $$A_{i}=\mathrm{hash}\left(\overset{m\times n\times 5}{\underset{x=1}{\sum }}\left(a_{x}\times 2^{\left(x-1\right)}\right)\right)$$

Step 5. The watermark $W_{i}$ that will be embedded into each block $B_{i}$ is generated using Equation (6).

(6) $$W_{i}=(R_{i}||A_{i})$$

Step 6. Finally, the watermark $W_{i}$ is embedded into the q-LSB of each block using the least significant bit (LSB) replacement method.

**Figure 2:**
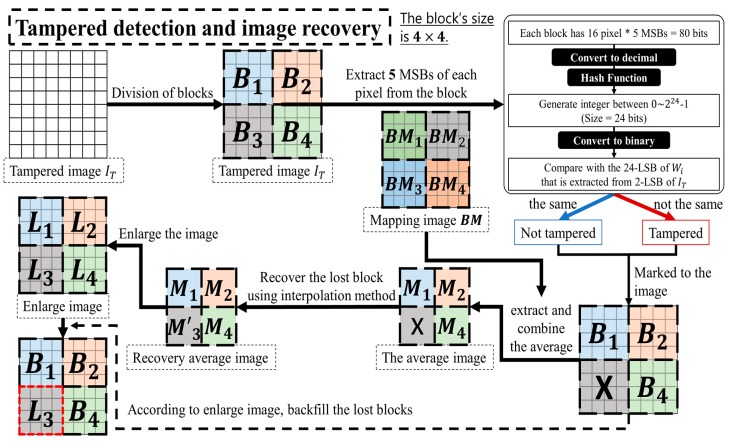
Flowchart of tamper detection and image recovery process based on “block-wise detection” (The size of a block is 4×4).

In the image tamper detection process, steps of generating authentication data are similar to the steps mentioned in the section above. These steps are used to obtain k-bit authentication data ${A^{\prime }}_{i}$ for block $B_{i}$. The authentication data $A_{i}$ that was embedded in block $B_{i}$ is then extracted from the tampered image $I_{T}$. Comparing the similarity of the authentication data $A_{i}$ that was embedded in Block $B_{i}$, to the re-calculated authentication data ${A^{\prime }}_{i}$, if ${A^{\prime }}_{i}=A_{i}$, indicates that the image was not tampered. On the other hand, if the extracted authentication data $A_{i}$ differs from the re-calculated authentication ${A^{\prime }}_{i}$ (${A^{\prime }}_{i}\neq A_{i}$), it indicates that the image block may have been tampered. We mark these image blocks as tampered areas. The flowchart of the tamper detection process is shown in Figure 2. The following steps provide a detailed description on tamper detection and image recovery methods.

Step 1. The tampered image $I_{T}$ is divided into non-overlapping block $B_{i}$. Each block’s size is m×n.

Step 2. The re-calculated authentication data ${A^{\prime }}_{i}$ of the block $B_{i}$ is generated by Equations (4) and (5) from $I_{T}$.

Step 3. The watermark $W_{i}$ is extracted from the q-LSB of the block $B_{i}$’s pixel $P_{j}^{i}$ in $I_{T}$. And using the function RightSubBit($W_{i},k$), the extracted authentication data $A_{i}$ is extracted from the right k bits of $W_{i}$.

Step 4. The re-calculated authentication data ${A^{\prime }}_{i}$ is compared with the extracted authentication data $A_{i}$. According to Equation (7), if the result is not the same, then $T_{i}=1$, and it represents this block as ‘a tampered block’. Otherwise, if the result is the same, then $T_{i}=0$, and it represents this block as ‘not a tampered block’.

(7) $$T_{i}=\{\begin{array}{c} 1,\;{A^{\prime }}_{i}\neq A_{i}\\ 0,\;{A^{\prime }}_{i}=A_{i} \end{array}$$

Step 5. The block $B_{i}$ and the tamper mark table $T_{i}$ generate the mapping block $BM_{i}$ and the mapping tamper mark table $TM_{i}$ using the secret key SK, respectively.

Step 6. For the mapping block $BM_{i}$ that is $TM_{i}=0$, the recovery data $R_{i}$ is extracted from the 8-MSB of the watermark $W_{i}$.

Step 7. If the tampered image $I_{T}$ is not tampered, then the extracted recovery data $R_{i}$ is the 1/(m×n) multiple images of the original image $I_{O}$. But if the image is tampered, then the extracted recovery data image will have lost pixels. Because the image texture is coherent, we can use the surrounding pixel values to fill in the lost pixels value by interpolation method.

Step 8. The enlarged image L is generated by Bicubic Interpolation function [36].

Step 9. The lost block ($T_{i}=1$) is filled by the corresponding block of the enlarged image L, and then the recovery image $I_{R}$ is generated.

### 3.3. Pixel-Wise Detection

The block-wise detection method was introduced in the previous section. As the authentication data is based on blocks, when tampered pixels are detected, the entire block to which the pixel belongs will be marked as tampered. However, if authentication data is embedded based on pixels, then only the tampered pixels will be marked as tampered when detected. Therefore, when embedding authentication data, the pixel-wise image authentication method is much more accurate than those based on the block-wise image authentication method. This section describes a data hiding technique based on the pixel-wise image authentication method.

**Figure 3:**
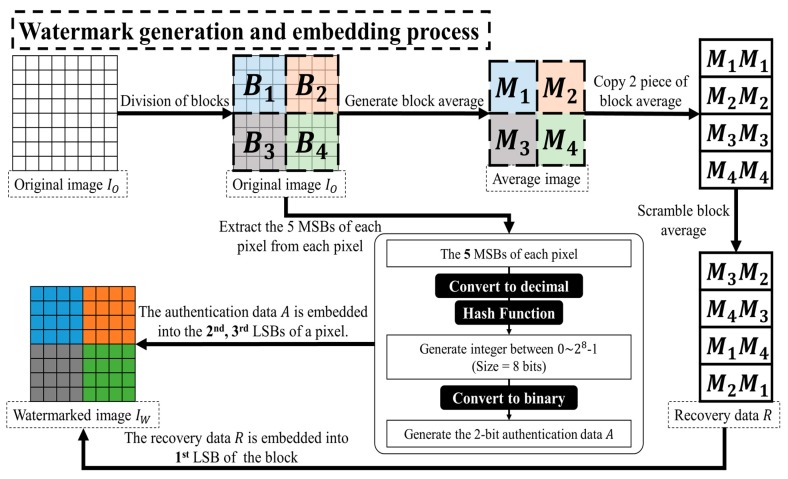
Flowchart of the watermark generation and embedding process based on “pixel-wise detection”.

The process of generating a watermark is similar to the method based on block detection. The flow chart is shown in Figure 3 and the following steps provide a detailed description of the watermark generation and the embedding process:

Step 1. The original image $I_{O}$ is divided into 4×4 non-overlapping blocks $B_{i}$.

Step 2. According to Equation (1), the mean value $M_{i}$ of each block $B_{i}$ is calculated. The mean value of each block has two copies, as in Equation (8). The non-repeating pseudo-random sequence $RM=\left\{rm_{1},rm_{2},\ldots ,rm_{N\times 2}\right\}$ is generated by the secret key SK. And two copies of the mean value are scrambled by Equation (9) as the recovery data R. The recovery data $R_{i}$ which will be embedded into each block $B_{i}$ is $\left[\begin{array}{cc} M_{rm_{i}} & M_{rm_{i+N}} \end{array}\right].$ The reason for embedding two copies of the block mean value in the image is that the survival chances of the recovery data may increase after the image has been maliciously tampered.

(8) $$M_{C}=\left[\begin{array}{c} \begin{array}{cc} M_{1} & M_{1}\\ M_{2} & M_{2}\\ \vdots & \vdots \\ M_{N} & M_{N} \end{array} \end{array}\right]$$

(9) $$R=\left[\begin{array}{c} \begin{array}{cc} M_{rm_{1}} & M_{rm_{N+1}}\\ M_{rm_{2}} & M_{rm_{N+2}}\\ \vdots & \vdots \\ M_{rm_{N}} & M_{rm_{N+N}} \end{array} \end{array}\right]$$

Step 3. According to Equation (10), the 8-bit encryption code $H_{j}^{i}$ is generated by hash function after extracting 5 most significant bits (MSBs) of each pixel value $P_{j}^{i}$. Then the 8th least significant bit (LSB), the 7th LSB, the 2nd LSB, the 1st LSB, and the 6th LSB, the 5th LSB, the 4th LSB, the 3rd LSB of the encryption code $H_{j}^{i}$ are calculated by exclusive or (XOR) operation, respectively. Thereafter, the authentication data $A_{j}^{i}=\left\{a_{1},a_{2}\right\}$ is generated.

(10) $$\{\begin{array}{c} p_{x}=\mathrm{mod}\left(P_{j}^{i}/2^{x},2\right)\;x=1,2,\ldots ,5\\ H_{j}^{i}=\mathrm{hash}\left(\overset{5}{\underset{x=1}{\sum }}\left(p_{x}\times 2^{\left(x-1\right)}\right)\right)\;\;\;\;\;\; \end{array}$$

Step 4. Finally, the recovery data R is embedded into 1st LSB of each pixel value in each block, and the authentication data $\left\{a_{1},a_{2}\right\}$ is embedded into the 2nd LSB and the 3rd LSB of the pixel, respectively.

In the tamper detection process, since authentication data is embedded based on pixels, the tampered pixels will be marked when any tamper is detected. Therefore, the result of tamper detection is much more accurate for the pixel-wise method compared to the block-wise method. The following steps provide a detailed description of tamper detection and self-recovery.

**Figure 4:**
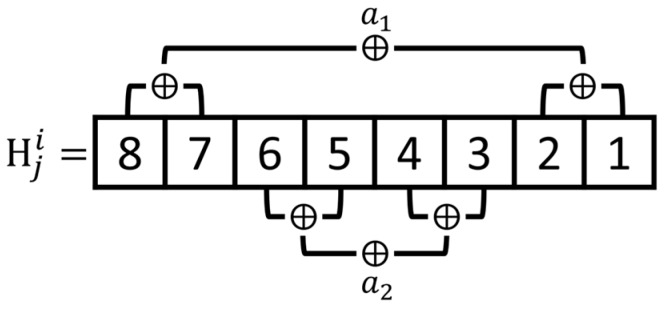
The authentication data $A=\left\{a_{1},a_{2}\right\}$ generation.

Step 1. Similar to the tamper detection and image recovery process described in Section 3.2, the re-calculation of authentication data ${A^{\prime }}_{j}^{i}$ is done from each pixel of the tampered image $I_{T}$ with reference to Equation (10) and Figure 4.

Step 2. The authentication data $A_{j}^{i}$ is extracted from the 2nd and the 3rd LSBs of each pixel $P_{j}^{i}$, and is compared with the re-calculated authentication data ${A^{\prime }}_{j}^{i}$. 

Step 3. If the extracted authentication data $A_{j}^{i}$ is the same as the re-calculated authentication data ${A^{\prime }}_{j}^{i}$, then the pixel is not tampered and $T_{i}=0$. In other cases, the pixel is tampered and $T_{i}=1$.

(11) $$T_{i}=\{\begin{array}{c} 1,\;{A^{\prime }}_{j}^{i}\neq A_{j}^{i}\\ 0,\;{A^{\prime }}_{j}^{i}=A_{j}^{i} \end{array}$$

Step 4. First, the entire image is divided into non-overlapping blocks. For $T_{i}=0$, the 1st LSB of each pixel in the block is extracted from the block of the non-tampered pixel, and combined as the recovery data $R_{i}$ of that block.

Step 5. Since the recovery data $R_{i}$ are the two copies of the block mean value that were disrupted, the recovery data is sorted using the secret key SK.

Step 6. If the number of the extracted mean value is less than half of the number of the original mean value, then skip to Step 8; otherwise, the tampered pixel is recovered from Step 7.

Step 7. The recovery data $R_{i}$ is combined as a smaller image, and the lost pixel value is filled by the interpolation method. The tampered pixel is corrected to the pixel of the smaller image.

Step 8. If the recovery data is incomplete, the tampered pixel cannot be recovered. So the mean value of the surrounding eight pixel values is calculated, and the tampered pixel is corrected to this value.

**Figure 5:**
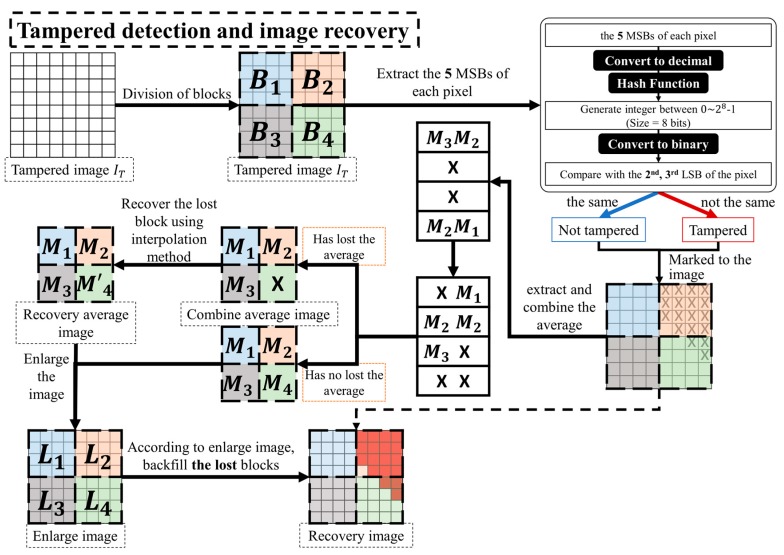
Flowchart of tamper detection and image recovery process based on “pixel-wise detection”.

Figure 5 displays the flowchart of tamper detection and image recovery process based on “pixel-wise detection.”

## 4. Experimental Results and Comparison

**Figure 6:**
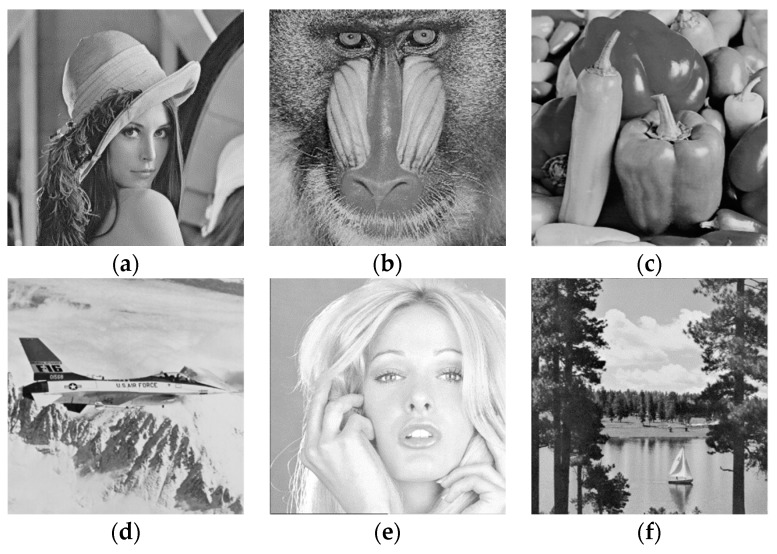
Six 512×512 test images. (**a**) Lena, (**b**) Baboon, (**c**) Peppers, (**d**) Airplane, (**e**) Tiffany, (**f**) Lake.

The experimental results of this paper are performed using MATLAB 2017a in a Windows 10, Intel Core i7 3.60 GHz system with 4 GB of memory. We used six grayscale images of size 512×512 (Lena, Baboon, Peppers, Airplane, Tiffany, Lake) as shown in the Figure 6.

For the measurement results of tampered detection, we evaluated the error rate of tampered detection by False Negative Rate (FNR) and False Positive Rate (FPR). The algorithm is shown in Equations (12) and (13). The error of tampered detection is divided into False Negative (FN) and False Positive (FP). The False Positive is the number of the pixel that is a non-tampered pixel but is detected as tampered; the False Negative is the number of pixels that are tampered but are detected as a non-tampered pixel. And TP (True Positive) denotes the number that are correctly detected for the tampered pixel, and TN (True Negative) denotes the number that are correctly detected for the untampered pixel [35].

(12) FNR=FN÷(FN+TP)

(13) FPR=FP÷(FP+TN)

The image quality measurement of the watermarked image and the recovered image uses peak signal to noise ratio (PSNR). As a result, if the image has a higher PSNR, the image quality is better. Their equations are represented as Equations (14) and (15), where H and W are the height and width of the image, and $I_{O}\left(i\right)$ and $I_{T}\left(i\right)$ are the ith pixel of the original image and the measured image.

(14) $$MSE=\frac{1}{H\times W}\overset{H\times W}{\underset{i=1}{\sum }}{\left(I_{O}\left(i\right)-I_{T}\left(i\right)\right)}^{2}$$

(15) $$PSNR=10\times log\frac{255^{2}}{\mathrm{MSE}}$$

In addition, we also use the Structural Similarity Index (SSIM) to measure the similarity between the original image and the watermarked image. The higher SSIM value indicates that the similarity between the original image and the watermark image is higher. The calculation method is shown in Equation (16), where $\mu_{x}$ and $\mu_{y}$ are the average values of the original image and the watermarked image, $\sigma_{xy}$ is the co-variation of the original image and the watermarked image, and $\sigma_{x}$ and $\sigma_{y}$ are the variation of the original image and the watermarked image, respectively. $C_{1}$ and $C_{2}$ are constants.

(16) $$SSIM=\frac{\left(2\mu_{x}\mu_{y}+C_{1}\right)\left(2\sigma_{xy}+C_{2}\right)}{\left(\mu_{x}^{2}+\mu_{y}^{2}+C_{1}\right)\left(\sigma_{x}^{2}+\sigma_{y}^{2}+C_{2}\right)}$$

### 4.1. Digital Image Tamper Detection

This section analyzes and explores embedding methods utilizing different block sizes. For the block-wise detection method, we used three different block sizes of 2×4, 3×3, and 4×4, to examine and compare error rates as well as image quality. The results obtained are as shown in Table 2. The tamper rate is set at 10%. The condition of q=3, k=16 is set for the 2×4 block size, which indicates that the watermark is embedded in the 3 LSBs of each pixel and the authentication data length is 16-bit. The false positive rate (FPR) is 0.17335% and the image quality of the watermarked and recovered images are PSNR^(w)^ = 41.28 dB and PSNR^(r)^ = 44.48 dB, respectively. Under the condition of q=3, k=16, the FPR is 0.13257% for 3×3 block size, which is lower than the FPR for 2×4 block size. The difference in the block size will affect FPR, as the entire block will be marked as a tampered block when a tampered pixel is detected using this method. The watermark image quality PSNR^(w)^ and recovered image quality PSNR^(r)^ for images divided into 3×3 blocks are slightly better than that of images divided into 2×4 blocks. Lastly, when the block size is 4×4 and the condition is set to q=2, k=24, the PSNR^(w)^ = 47.32 dB, which is much higher than the other two block sizes; and PSNR^(r)^ = 44.68 dB, which is slightly higher when compared to the other two block sizes. This may be because the watermark is only embedded in the 2 LSBs of a pixel. Overall, the quality of the recovered images PSNR^(r)^ are similar to each other, which is about 44 dB.

**Figure 7:**
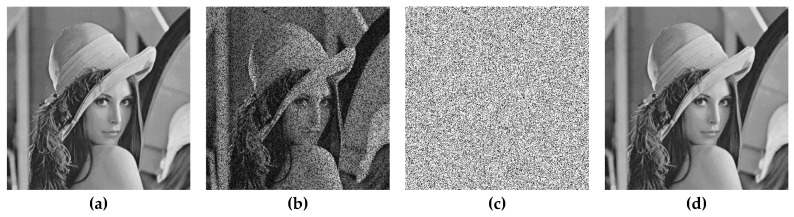
Watermarked Lena and tamper detection results using 4×4 pixel-wise detection (**a**) Watermarked image (${\mathrm{PSNR}}^{\left(w\right)}=41\;\mathrm{dB}$ ), (**b**) tampered image (Tamper rate = 30 %), (**c**) tamper detection result (FPR=0%), (**d**) recovery image (${\mathrm{PSNR}}^{\left(r\right)}=40.68\;\mathrm{dB}$ ).

**Figure 8:**
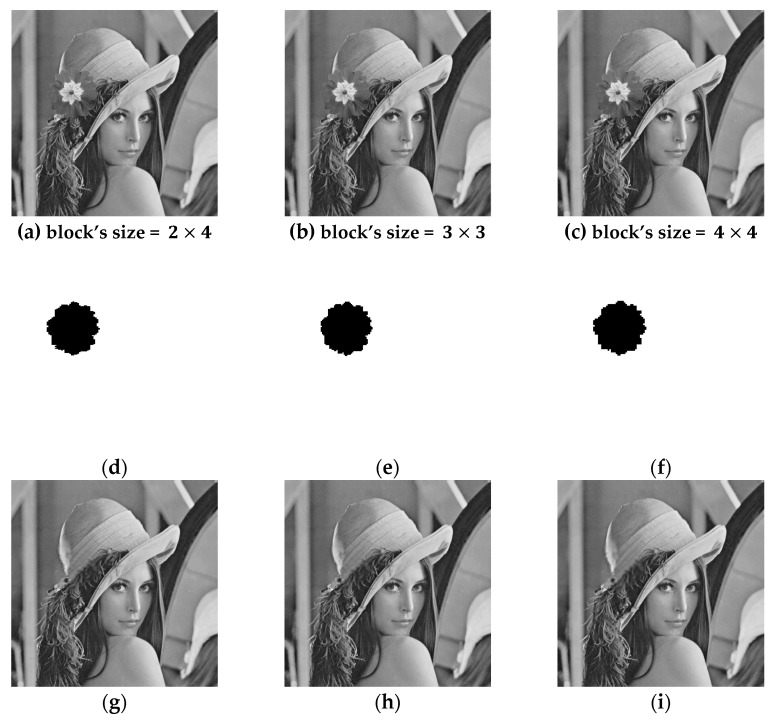
Watermarked Lena and tamper detection results for different block sizes using “block-size detection” (**a**–**c**) Tampered images (tamper rate = 4.4%), (**d**–**f**) tamper detection result, (**g**–**i**) recovery images.

**Figure 9:**
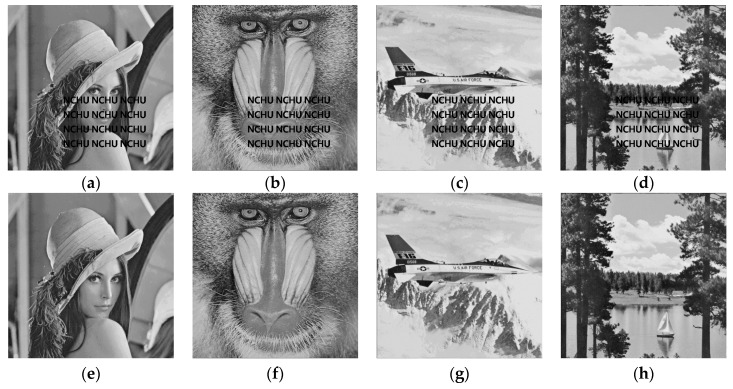
Tamper detection results using “pixel-wise detection” (The test images are Lena, Baboon, Airplane and Lake) (**a**–**d**) Tampered images (tamper rate = 3.7%), (**e**–**h**) recovery images.

Figure 7 shows the results of tamper detection and recovery from salt and pepper noise attack. Figure 7c is the tamper detection result, which shows no error in detection. Figure 7d is the result of the recovered image, which shows that its image quality, PSNR^(r)^, is 40.68 dB. In Figure 8, a flower is added on top of Lena’s hat, and the block-wise detection method was used. Figure 8a–c shows tampered images with a tamper rate of 4.4%. Figure 8d–f shows the detection results, and Figure 8g–i shows recovered images. The quality of recovered images is all above 45 dB. Figure 9 indicates the tamper detection results using 4×4 blocks. Figure 9a–d are tampered images of Lena, Baboon, Airplane, and Lake, with a tamper rate of 3.7%. Figure 9e–h are the results of image recovery, and the image quality PSNR^(r)^ are all above 45 dB. It is evident that our proposed image authentication method could resist diverse tampering attacks.

The block-wise detection method that we proposed uses a 4×4 block size. Each image’s PSNR and error rate for different tamper rates are shown in Table 3. Since watermark authentication data are embedded in the 2 LSBs of a pixel, the PSNR^(w)^ of every image is higher than 47 dB. When the tamper rate is 10%, the PSNR^(r)^ of the recovered image is close to 50 dB for Lena’s image. At a high tamper rate of 50%, the recovered image PSNR^(r)^ of each image is still above 32 dB. Airplane and Tiffany images are the best performing images with a PSNR^(r)^ = 36.79 dB. With respect to error rate, since our method uses blocks to detect tampered blocks, the error rate is lesser subjected to the effects of higher tamper rates when clipping attacks occur.

### 4.2. Comparison with Other Methods

Table 4 shows a comparison of efficiency between our method and [21,22,24,31,32] for maximum tampering rate using the Lena image. For the methods proposed by Yang and Shen [21], Yang et al. [22], and Kim et al. [32], the maximum tolerable tamper rate is less than 50%, which is the same as our methods. But the PSNR^(r)^ values for block-wise method and pixel-wise method of our methods are 36.62 dB and 37.26 dB, which is higher than those of Yang and Shen’s [21], Yang et al.’s [22] and Kim et al.’s [32] methods. In addition, the PSNR^(r)^ of Qin et al. [31] is higher than 3.74 dB compared to our method based on pixel-wise detection method, but their maximum tolerable tamper rate is less than 45%. Table 5 shows the SSIM performance of the proposed method using six standard testing images. The results show that the block-wise method has better structure similarity for image Baboon and least similarity for image Tiffany. As for the pixel-wise method, image Baboon still has better similarity, and image Peppers has the least similarity when using the pixel-wise method. Overall, the 4×4 block-wise method has the best SSIM performance at 0.986461 and the worst SSIM at 0.955061. Table 6 shows a comparison of the false positive rate (FPR) and the false negative rate (FNR) of the proposed methods and the pervious methods [25,26,28,34] using several test images.

**Figure 10:**
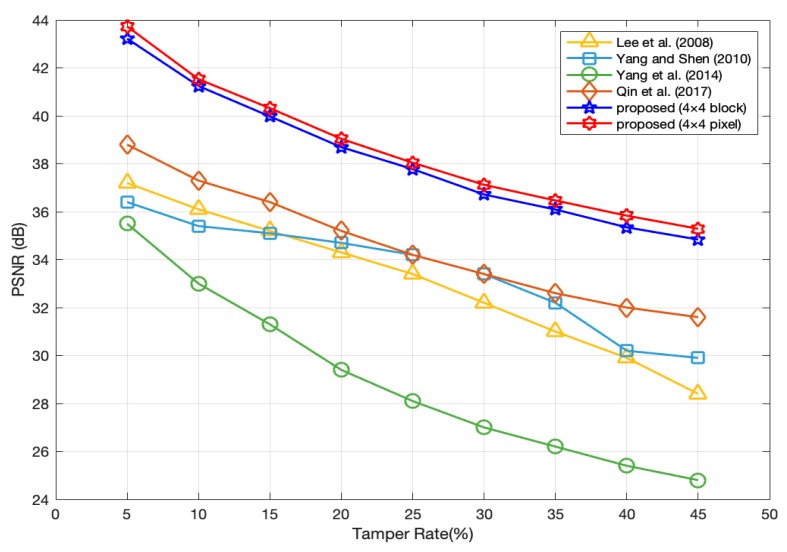
Peak signal to noise ratio (PSNR^(r)^) comparison of the recovered image between the proposed methods and methods in [20,21,22,31].

The comparison of recovered image quality are shown in Figure 10 under different tamper rates among our proposed methods and other fragile watermarking methods such as Lee et al. [20], Yang and Shen [21], Yang et al. [22], Qin et al. [31]. From this, it is evident that for our method, the PSNR of recovered images is about 2 dB higher than that of other methods at any tamper rate. Therefore, in terms of image recovery, the performance of our method is excellent.

Table 7 shows the performance of the proposed method in terms of FPR, FNR, and recovered image quality when dealing with complex images. Baboon was used to represent these kinds of image attacks. According to Table 7, even if 14.35% of Baboon was tampered its recovered PSNR is still above 38 dB, and with 4×4 block size the PSNR by pixel-wise approach gets even higher to 39.21 dB. Furthermore, after examining several complex images, the FNR was always found to be 0, which means that the area which has been tampered will always be detected. 

Table 8 shows the results of 500 test images of the size 512×512 using the proposed block-wise method. It can be seen that the FPR and FNR values become lower or even approached to zero when the block size reduces. The highest values of FNR and FPR were always below 0.000082 and 0.038, respectively. In addition, the recovered $PSNR_{R}$ value was always above 36 dB. 

Table 9 shows the comparison of the processing complexity of each method. Images of size 256×256 were used to examine each method. These watermarking schemes contain block types, authentication information, and recovery information. Each method’s processing steps are displayed in terms of block division, discrete wavelet transform (DWT), DWT embedding, discrete cosine transform (DCT), mean calculation, watermark hashing, Hilbert curve transformation, exclusive or (XOR), least significant bit (LSB) embedding, and histogram shifting and modification. The total number of steps in the watermarking procedure are also presented for each method. In Table 9, there will be higher operation counts for pixel-wise methods, such as Lo and Hu [27], Qin et al. [31] and the proposed pixel-wise method. The methods of Hsu and Tu [23], Yin et al. [30], Singh and Singh [29], Tai and Liao [33] are block-wise. Among them, the proposed block-wise method has the least operation count. 

## 5. Conclusions

In this paper, we proposed a self-recovery fragile watermarking image authentication technology for wireless sensor networks. The authentication methods are classified into two types: block-wise and pixel-wise methods. In the block-wise detection method, authentication data is generated from each block, and the average block value is used to generate recovery data. Further, the length of authentication data and recovery data are adjusted according to the size of each block. From the experimental results, it can be seen that when a tampered pixel is detected, the block to which it belongs, will be marked as a tampered block. Therefore, if the block division is small, the false positive rate (FPR) will be small as well. In the pixel-wise detection method, the authentication data is generated from each pixel, and the recovery data is generated from the mean value of the 4×4 block. When the tamper rate is 50%, the PSNR of the recovered image of all tested images is above 32 dB. Compared with other methods, our method has a better performance for tamper detection and image recovery.

Using our proposed method, future research should attempt to use different extraction techniques that demonstrate characteristics of the block during the process of generating recovery data, such as the absolute moment block truncation coding (AMBTC) method. In addition to the common clipping and peppered salt attacks, we did not conduct relevant vector quantization attack experiments with respect to tamper experiments. Future research can consider them for in-depth studies. In addition, our method belongs to the fragile watermarking method. After using common image processes, such as JPEG compression, the recovery and authentication data embedded in the image get destroyed, and tampered areas cannot be detected accurately. But JPEG compression can reduce the need of transmitting bandwidth effectivity in WSNs. Therefore, authentication robustness in image compression is also a topic of concern which can be explored in further studies.

## Figures and Tables

**Table 1:** Relationship between the original block and the mapping block.

i	1	2	3	4	…	N
SK **generation**	↓	↓	↓	↓	↓	↓
$rm_{i}$	N	4	1	2		3
$B_{i}$	$B_{1}$	$B_{2}$	$B_{3}$	$B_{4}$	…	$B_{N}$
**Associated blocks**	↓	↓	↓	↓	↓	↓
$B_{rm_{i}}$	$B_{N}$	$B_{4}$	$B_{4}$	$B_{2}$		$B_{3}$
$BM_{i}$	$BM_{1}$	$BM_{2}$	$BM_{3}$	$BM_{4}$		$BM_{N}$

**Table 2:** Comparison of the average error and image quality for block-wise detection.

m	n	q	k	FPR (%)	PSNR^(w)^ (dB)	PSNR^(r)^ (dB)
2	4	3	16	0.17335	41.28	44.48
3	3	3	16	0.13257	41.77	44.51
4	4	2	24	0.17335	47.32	44.68

**Table 3:** Comparison of the 4×4 “block-wise detection”.

Image	PSNR^(w)^ (dB)	Tamper Rate	10%	20%	30%	40%	50%
Lena	47.20	PSNR^(r)^ (dB)	49.47	44.39	41.23	38.58	36.61
		FNR (%)	0	0	0	0	0
		FPR (%)	0.173	0.391	0.669	0.787	0
Baboon	47.29	PSNR^(r)^ (dB)	38.69	35.55	33.95	32.93	32.13
		FNR (%)	0	0	0	0	0
		FPR (%)	0.173	0.391	0.669	0.787	0
Peppers	47.23	PSNR^(r)^ (dB)	42.84	40.54	38.32	36.76	35.17
		FNR (%)	0	0	0	0	0
		FPR (%)	0.173	0.391	0.669	0.787	0
Airplane	47.33	PSNR^(r)^ (dB)	46.59	44.54	42.83	40.32	36.79
		FNR (%)	0	0	0	0	0
		FPR (%)	0.173	0.391	0.669	0.787	0
Tiffany	47.54	PSNR^(r)^ (dB)	45.81	41.87	40.08	38.36	36.79
		FNR (%)	0	0	0	0	0
		FPR (%)	0.173	0.391	0.669	0.787	0

**Table 4:** Performance comparisons of proposed methods and [21,22,24,31,32].

Methods	PSNR^(w)^ (dB)	PSNR^(r)^ (dB)	Condition of Restoration
Yang and Shen [21]	40.7	32	<50%
Yang et al. [22]	51.3	36	<50%
Qian et al. [24]	37.9	35	<35%
Qin et al. [31]	46	41	<45%
Kim et al. [32]	43.7	33.6	<50%
Proposed method (4×4 block-wise detection)	43.73	36.62	<50%
Proposed method (4×4 pixel-wise detection)	41	37.26	<50%

**Table 5:** Structural Similarity Index (SSIM) performance of proposed methods.

	2×4 Block-Wise	3×3 Block-Wise	4×4 Block-Wise	4×4 Pixel-Wise
512_lena	0.92739	0.93511	0.958791	0.94159
512_baboon	**0.97581**	**0.97843**	**0.986461**	**0.97489**
512_peppers	0.93037	0.93739	0.961136	**0.93938**
512_airplane	0.92725	0.93173	0.95682	0.94193
512_tiffany	**0.91812**	**0.92558**	**0.955061**	0.94091
512_lake	0.94736	0.95261	0.970439	0.96453

**Table 6:** False positive rate (FPR) and false negative rate (FNR) comparison of proposed methods and [25,26,28,34].

Methods	FPR(%)	FNR(%)
Tong et al. [25]	0.22	0
Chen et al. [26]	0.25	0
Ansari et al. [28]	0.5	0.01
Wang et al. [34]	0.30	0
Proposed method (4×4 block-wise detection)	0.173	0
Proposed method (4×4 pixel-wise detection)	0	0

**Table 7:** Recovered PSNR^(r)^, FPR, and FNR results of the proposed method with complex images.

	2×4 Block-Wise	3×3 Block-Wise	4×4 Block-Wise	4×4 Pixel-Wise
**Tamper Rate (%)**	14.35	14.35	14.35	14.35
**Recovered PSNR^(r)^ (dB)**	38.749796	38.855165	38.192091	39.2149
**FNR(%)**	0	0	0	0
**FPR(%)**	0.029	0.025	0.038	0

**Table 8:** Recovered PSNR, FPR, and FNR results of the proposed method (block-wise) using 500 test images under the tamper rate of 14.35% with variant block sizes.

Block size		2 × 4	3 × 3	4 × 4
**Tamper Rate (%)**		14.35	14.35	14.35
**Recovered PSNR^(r)^**	average	41.55	41.54	40.61
	highest	**47.55**	**48.09**	**48.18**
	lowest	37.45	37.41	36.77
**FNR(%)**	average	0.000082	0.000274	0.000562
	highest	0.000824	0.003803	0.006887
	lowest	**0.000000**	**0.000000**	**0.000000**
**FPR(%)**	average	0.02850	0.02531	0.03764
	highest	0.02855	0.02548	0.03803
	lowest	**0.02801**	**0.02324**	**0.03526**

**Table 9:** Processing complexity comparison.

	Hsu and Tu [23]	Lo and Hu [27]	Singh & Singh [29]	Yin et al. [30]	Qin et al. [31]	Tai and Liao [33]	Proposed Block-Wise	Proposed Pixel-Wise
Block Size	8×8	4×4	2×2	4×4	3×3	4×4	4×4	4×4
Block division	5120	4096	16,384	4096	64,516	4096	4096	4096
Predictive coding and it inverse	0	65,536	0	0	0	0	0	0
Arnold’s permutation	0	0	0	0	0	4096	0	0
DWT	4096	0	0	16,384	0	4096	0	0
DWT embedding	0	0	0	0	0	4096	0	0
DCT	0	0	16,384	0	0	0	0	0
Mean calculation	4096	0	16,384	0	0	0	4096	4096
Watermark hashing	1024	0	16,384	0	64,516	4096	4096	65,536
Hilbert curve transformation	1024	0	0	4096	64,516	0	0	0
XOR	20,480	0	0	4096	0	4096	4096	65,536
LSB embedding	1024	0	16,384	0	0	0	4096	65,536
Histogram shifting and modification	1024	65,536	16,384	4096	64,516	0	0	0
Total operation count	37,888	139,264	81,920	32,768	258,064	62,464	20,480	204,800

## References

[1] Feng J., Potkonjak M. (2003). Real-time watermarking techniques for sensor networks. Proc. SPIE.

[2] Wong J.L., Feng J., Kirovski D., Potkonjak M., Raghavendra C.S., Sivalingam K.M., Znati T. (2004). Security in sensor networks: Watermarking techniques. Wireless Sensor Networks.

[3] Shi X., Xiao D. (2013). A reversible watermarking authentication scheme for wireless sensor networks. Inf. Sci..

[4] Singh A.K., Kumar B., Dave M., Mohan A. (2015). Robust and imperceptible dual watermarking for telemedicine applications. Wirel. Pers. Commun..

[5] Lou D.-C., Liu J.-L. (2000). Fault resilient and compression tolerant digital signature for image authentication. IEEE Trans. Consum. Electron..

[6] Tsai P., Hu Y., Chang C. (2005). Novel image authentication scheme based on quadtree segmentation. Imaging Sci. J..

[7] Ababneh S., Ansari R., Khokhar A. (2009). Iterative compensation schemes for multimedia content authentication. J. Vis. Commun. Image Represent..

[8] Umamageswari A., Suresh G.R. (2014). Secure medical image communication using ROI based lossless watermarking and novel digital signature. J. Eng. Res..

[9] Das C., Panigrahi S., Sharma V.K., Mahapatra K.K. (2014). A novel blind robust image watermarking in DCT domain using inter-block coefficient correlation. AEU Int. J. Electron. Commun..

[10] Mishra A., Agarwal C., Sharma A., Bedi P. (2014). Optimized gray-scale image watermarking using DWT-SVD and firefly algorithm. Expert Syst. Appl..

[11] Parah S.A., Sheikh J.A., Loan N.A., Bhat G.M. (2016). Robust and blind watermarking technique in DCT domain using inter-block coefficient differencing. Digit. Signal Process..

[12] Di Y.-F., Lee C.-F., Wang Z.-H., Chang C.-C., Li J. (2016). A robust and removable watermarking scheme using singular value decomposition. KSII Trans. Internet Inf. Syst..

[13] Singh A.K. (2017). Improved hybrid algorithm for robust and imperceptible multiple watermarking using digital images. Multimed. Tools Appl..

[14] Huynh-The T., Hua C.-H., Anh Tu N., Hur T., Bang J., Kim D., Bilal Amin M., Kang B.H., Seung H., Lee S. (2018). Selective bit embedding scheme for robust blind color image watermarking. Inf. Sci..

[15] Zear A., Singh A.K., Kumar P. (2018). A proposed secure multiple watermarking technique based on dwt, DCT and SVD for application in medicine. Multimed. Tools Appl..

[16] Lee C.-F., Chen H.-L., Yang T.-C. (2011). Semi-blind watermarking scheme exploiting self-reference image. J. Internet Technol..

[17] Preda R.O. (2013). Semi-fragile watermarking for image authentication with sensitive tamper localization in the wavelet domain. Measurement..

[18] Al-Otum H.M. (2014). Semi-fragile watermarking for grayscale image authentication and tamper detection based on an adjusted expanded-bit multiscale quantization-based technique. J. Vis. Commun. Image Represent..

[19] Qi X.J., Xin X. (2015). A singular-value-based semi-fragile watermarking scheme for image content authentication with tamper localization. J. Vis. Commun Image Represent..

[20] Lee T.-Y., Lin S.D. (2008). Dual watermark for image tamper detection and recovery. Pattern Recogn..

[21] Yang C.-W., Shen J.-J. (2010). Recover the tampered image based on VQ indexing. Signal. Process..

[22] Yang S., Qin C., Qian Z., Xu B. Tampering detection and content recovery for digital images using halftone mechanism. Proceedings of the 2014 Tenth Intelligent Information Hiding and Multimedia Signal Processing (IIH-MSP 2014).

[23] Hsu C.-S., Tu S.-F. (2016). Image tamper detection and recovery using adaptive embedding rules. Measurement.

[24] Qian Z., Feng G., Zhang X., Wang S. (2011). Image self-embedding with high-quality restoration capability. Digit. Signal Process..

[25] Tong X.J., Liu Y., Zhang M., Chen Y. (2013). A novel chaos-based fragile watermarking for image tampering detection and self-recovery. Signal Process. Image Commun..

[26] Chen F., He H.J., Tai H.M., Wang H.X. (2014). Chaos-based self-embedding fragile watermarking with flexible watermark payload. Multimed. Tools Appl..

[27] Lo C.-C., Hu Y.-C. (2014). A novel reversible image authentication scheme for digital images. Signal Process..

[28] Ansari I.A., Pant M., Ahn C.W. (2016). SVD based fragile watermarking scheme for tamper localization and self-recovery. Int. J. Mach. Learn. Cybern..

[29] Singh D., Singh S.K. (2016). Effective self-embedding watermarking scheme for image tampered detection and localization with recovery capability. J. Vis. Commun Image Represent..

[30] Yin Z., Niu X., Zhou Z., Tang J., Luo B. (2016). Improved reversible image authentication scheme. Cogn. Comput..

[31] Qin C., Zhang P., Ji X., Dong J., Wang J. (2017). Fragile image watermarking with pixel-wise recovery based on overlapping embedding strategy. Signal Process..

[32] Kim C., Shin D., Yang C.-N. (2018). Self-embedding fragile watermarking scheme to restoration of a tampered image using AMBTC. Pers. Ubiquit. Comput..

[33] Tai W.-L., Liao Z.-J. (2018). Image self-recovery with watermark self-embedding. Signal Process..

[34] Wang C., Zhang H., Zhou X. (2018). A Self-recovery fragile image watermarking with variable watermark capacity. Appl. Sci..

[35] Katzenbeisser S., Petitcolas F.A.P. (2000). Information Hiding Techniques for Steganography and Digital Watermarking (Artech House Computer Security Series).

[36] Keys R. (1981). Cubic convolution interpolation for digital image processing. IEEE Trans. Acoust. Speech Signal Process..

